# Comparison of Updated Methods for *Legionella* Detection in Environmental Water Samples

**DOI:** 10.3390/ijerph18105436

**Published:** 2021-05-19

**Authors:** Daniela Toplitsch, Sabine Platzer, Romana Zehner, Stephanie Maitz, Franz Mascher, Clemens Kittinger

**Affiliations:** Diagnostic & Research Institute of Hygiene, Microbiology and Environmental Medicine, Medical University of Graz, 8010 Graz, Austria; daniela.toplitsch@medunigraz.at (D.T.); sabine.platzer@medunigraz.at (S.P.); romana.zehner@edu.uni-graz.at (R.Z.); stephanie.maitz@medunigraz.at (S.M.); franz.mascher@medunigraz.at (F.M.)

**Keywords:** risk assessment, water monitoring, water quality, bacteriological, *Legionella*

## Abstract

The difficulty of cultivation of *Legionella* spp. from water samples remains a strenuous task even for experienced laboratories. The long incubation periods for *Legionellae* make isolation difficult. In addition, the water samples themselves are often contaminated with accompanying microbial flora, and therefore require complex cultivation methods from diagnostic laboratories. In addition to the recent update of the standard culture method ISO 11731:2017, new strategies such as quantitative PCR (qPCR) are often discussed as alternatives or additions to conventional *Legionella* culture approaches. In this study, we compared ISO 11731:2017 with qPCR assays targeting *Legionella* spp., *Legionella pneumophila*, and *Legionella pneumophila* serogroup 1. In samples with a high burden of accompanying microbial flora, qPCR shows an excellent negative predictive value for *Legionella pneumophila*, thus making qPCR an excellent tool for pre-selection of negative samples prior to work-intensive culture methods. This and its low limit of detection make qPCR a diagnostic asset in Legionellosis outbreak investigations, where quick-risk assessments are essential, and are a useful method for monitoring risk sites.

## 1. Introduction

Though *Legionella* spp. exist ubiquitously in natural water environments and as a pollutant in artificial water systems, isolation of the elusive opportunistic pathogen *Legionella pneumophila* (*L. pneumophila*) remains a strenuous task [1]. *L. pneumophila* serogroup (sg) 1 is the most common infectious agent in Legionnaires Disease and in its milder form (Pontiac Fever) in Europe [2]), however, other *Legionella* spp. are also known to cause disease [3]. *Legionella* isolation requires laboratories with long experience in cultivation of the organism, as various factors such as the complex steps necessary for culture and competing microbial flora in the sample can influence culture accuracy [4,5,6]. The first differentiation of isolates of *Legionella* spp. from other bacteria happens by selection for L-cysteine auxotrophy via buffered charcoal yeast extract (BCYE) agar, containing cysteine and iron salts, as well as α-ketoglutarate [7]. The slow growth of the *Legionella* spp. isolates further necessitates the elimination of competing microbial flora, using harsh methods, such as heat and acid treatment, which are also thought to have a negative impact on the cultivability of the *Legionellae* and might lead to significant losses [6,7]. Additionally, it was shown that culture according to ISO 11731 is not very sensitive to the detection of *Legionella* non-*pneumophila* [4,8]. Culture requires long incubation periods of up to ten days, which constitute a problem in time-sensitive cases such as outbreak situations [2,5,7]. The high tolerance of *Legionella* to biocides, heat, and even acid, and its ability to persist, makes the constant monitoring of risk sources, such as cooling towers, hot, and cold-water systems or spa pools, essential [9,10].

All of the above-mentioned difficulties lead various scientists to the development of new methods for the detection and quantification of *Legionella* in water samples, one of the techniques suggested by numerous scientists being quantitative polymerase chain reaction (qPCR) [2,11,12]. Different assays were developed for this purpose, with some relying on intercalating fluorescent dyes such as SYBR Green for quantification, and others using molecular hybridization probe-based detection methods, such as TaqMan assays [13]. These methods enable the detection and quantification via the total DNA isolated from the sample, thus allowing precise quantification of low amounts of target gene [13]. The standard method ISO/TS 12869:2012-Water quality—detection and quantification of *Legionella* spp. or *Legionella pneumophila* by concentration and genic amplification by quantitative polymerase chain reaction (qPCR) was published as the reference guideline for laboratories using qPCR-based detection methods.

In order to improve the detection in the culture-based method, an updated version of the standard method ISO 11731:2017-*Water quality and Enumeration of Legionella*, referenced in most guidelines for drinking water, cooling towers, etc., implementing a decision matrix for sample analysis, was recently released. This reference method for detection of *Legionella* proposes three different methods for analyzing water samples, depending on the accompanying microbial flora in the water samples. One method, described as Matrix A, was recommended for the analysis of samples with low accompanying microbial flora, such as potable water. Matrix B was recommended for the analysis of samples with high accompanying microbial flora, such as cooling towers, cooling water, etc. Matrix C was recommended for samples with extremely high levels of accompanying flora, such as sewage.

Our study aimed at comparing the results for the detection of *Legionella* in water samples (detection of *Legionella* spp., *L. pneumophila* as well as *L. pneumophila* sg 1) obtained from the updated ISO 11731:2017, with results obtained by qPCR based on ISO/TS 12869:2012 for two different sample groups (samples with presumed low or high accompanying microbial flora), to gain further insights into their compatibility and usefulness in water sample valuation, for the difficult-to-handle *Legionella*. 

## 2. Materials and Methods

### 2.1. Water Samples

From March to May 2018, routine water samples (*n* = 64) screened for *Legionella* contamination were collected from the water laboratory at the Institute for Hygiene, Microbiology and Environmental Medicine at the Medical University of Graz. The samples were of mixed origin, with one category (*n* = 46) being samples with presumably low accompanying bacterial flora, such as water supply samples (*n* = 32) and water circuit samples of bathing water (*n* = 14), and the other category being samples with an expected high accompanying bacterial flora (*n* = 18), coming from cooling towers (*n* = 3), cooling water (*n* = 6), car wash facilities (*n* = 7), and system water (*n* = 2). All were analyzed for possible *Legionella* contamination by culture, as well as by qPCR.

### 2.2. Sample Preparation and DNA Extraction

Samples were collected in multiple, sterile 100 mL plastic bottles (VWR International, Vienna, Austria), 500 mL aliquots were used for quantification by culture, and 50–100 mL aliquots, depending on the amount of sample sent to the water laboratory, were used for quantification by qPCR. Samples were either analyzed immediately after arrival in the laboratory or within 24 h after arrival with storage at 4 °C, until preparation. For DNA extraction, aliquots were filtered through a 45 mm polycarbonate membrane with a 0.2 µm pore size (Isopore™ Membrane Filters, Merck Millipore Ltd., Darmstadt, Germany). Filters were stored until DNA extraction at −80 °C. DNA extraction was performed using the Qiagen PowerWater Kit (QIAGEN GmbH, Hilden, Germany), according to the manufacturer’s protocol. The quantity and purity of the DNA extracts were measured by the NanoDrop2000 instrument (Thermo Fisher Scientific Inc., Waltham, MA, USA). 

### 2.3. Quantification by Culture

The quantification of *L. pneumophila* sg 2–15 and sg 1 by culture was performed according to ISO 11731:2017. Sample handling was performed according to the decision matrix described in the standard method ISO 11731:2017 (see Table 1), with the exception of applying Matrix A as well as Matrix B, regardless of presumed accompanying microbial flora to compare both. No samples requiring Matrix C were included in this study.

For water supply samples and water circuit samples (presumed low burden of accompanying microbial flora), samples were analyzed using Matrix A as well as Matrix B. In brief, for Matrix A, 1 mL as well as 100 mL of the sample were filtered through a 47 mm mixed cellulose esters filter with a 0.45 µm pore size (EZ-Pak^®^ Membrane Filters, Millipore, Darmstadt, Germany). The filters were then directly placed on BCYE agar plates (VWR International, Vienna, Austria) (Method A). For Method B, 1 mL and 100 mL of the samples were filtered and microorganisms were subsequently recovered from the membrane filters using 5 mL of 2.5% Ringer’s solution, by vortexing and 0.250 mL of the rinsate were plated on GVPC agar (VWR International, Vienna, Austria) or the filters were treated with acid buffer (30 mL 0.2 mol·L^−1^ hydrochloric acid and 0.2 mol·L^−1^ potassium chloride acid solution; pH level of 2.2) for 5 min and then rinsed with 20 mL 2.5% Ringer’s solution, and the filters were then placed on GVPC agar (VWR International, Vienna, Austria). For heat treatment, 1 mL rinsate was heated for 30 min at 50 °C and 0.25 mL were then plated on GVPC agar (VWR International, Vienna, Austria).

For Matrix B, 100 mL of the sample were filtered through a 47-mm polycarbonate filter with a 0.2 µm pore size (Supor^®^ Membrane Filters, Pall Corporation, Dreieich, Germany), and microorganisms were subsequently recovered from the membrane filters, using 5 mL of 2.5% Ringer’s solution through vortexing. A total of 0.25 mL of the recovered sample were then plated on the GVPC agar plates (VWR International, Vienna, Austria). For heat treatment, another 0.25 mL of the recovered sample was incubated for 30 min at 50 °C and then plated on GVPC agar plates (VWR International, Vienna, Austria). For acid treatment, another 0.25 mL of the recovered sample was filtered through a 47-mm mixed cellulose esters filter with a 0.45 µm pore size (EZ-Pak^®^ Membrane Filters, Millipore, Darmstadt, Germany), which was then subjected to the acid buffer treatment, as described above. Afterwards, filters were rinsed with 20 mL of 2.5% Ringer’s solution. The rinsed filters were then placed on the GVPC agar plates (VWR International, Vienna, Austria).

For the cooling tower, cooling water, car wash facility water, system water, and bath water samples (presumed high burden of accompanying microbial flora), the samples were also analyzed using Matrix A and B, with the following modifications. For Matrix A, 0.01 mL, 0.1 mL, 1 mL, and 100 mL of the sample were filtered through a 47-mm mixed cellulose esters filter with a 0.45-µm pore size (EZ-Pak^®^ Membrane Filters, Millipore, Darmstadt, Germany) and was handled as described above. Matrix B was performed in the same manner as for the other sample types.

All plates were incubated at 36 °C for 7 to 10 days in a box (GENbox, bioMérieux, Vienna, Austria), and the colonies were counted at the end of the incubation period. Five or more presumptive *Legionella*-colonies were confirmed to be *Legionella* spp. by sub-culturing on Columbia blood agar plates (bioMérieux, Vienna, Austria) as well as the BCYE agar plates (VWR International, Vienna, Austria). The colonies were defined as *Legionella* spp. if no growth on cysteine-free Columbia blood agar plates, but growth on the BCYE agar plates occurred.

*Legionella* colonies growing on the BCYE agar plates (VWR International, Vienna, Austria) were further differentiated via latex agglutination testing in *L. pneumophila* sg 1, *L. pneumophila* sg 2–14, as well as *Legionella non-pneumophila* (*L. longbeachae* sg 1 and 2, *L. bozemanii* sg 1 and 2, *L. dumoffii*, *L. gormanii*, *L. jordanis*, *L. micdadei*, and *L. anisa*), using the LEGIONELLA LATEX TEST (Oxoid Deutschland GmbH, Vienna, Austria), according to the manufacturer’s instructions. For the final enumeration of *Legionella*, plates with the highest count of confirmed *Legionella* colonies were used. 

### 2.4. Quantification by Legionella-Specific qPCR

#### 2.4.1. qPCR Primers and Probe Sets

Primer and probe sets specific for *ssrA*, *mip*, *wzm*, and *egfp* (all primers as well as probes for *mip*, *wzm*, and *egfp* from Eurofins Genomics, Ebersberg, Germany; probe for *ssrA* from Applied Biosystems^®^, Warrington, Cheshire, UK) were selected from the current literature and used as previously described by Collins et al., 2015 [2] and Bliem et al., 2015 [14], with modification of fluorescent dyes or fluorescence quenchers (see Table 2). All gene targets occurred as a single copy in the *Legionella* genome.

#### 2.4.2. qPCR Conditions

Quantitative PCR was carried out according to ISO/TS 12869:2012, Collins et al., 2015 [2] and Toplitsch et al., 2018 [15], with the exception of using a simplex assay for each gene. For all reactions, the Luna^®^ Universal Probe qPCR Master Mix (New England Biolabs^®^ Inc., Frankfurt am Main, Germany) was used, and the reactions were carried out in a 20-µL reaction mix containing 400 nmol·L^−1^ of each *Legionella*-specific primer (600 nmol·L^−1^ primer for 5′-GGCGACCTGGCTTC-3′ for *ssrA*), 200 nmol·L^−1^ of the IAC plasmid primers, 150 nmol·L^−1^ of each *Legionella* specific probe, 75 nmol·L^−1^ IAC probe, and 0.4 µg/µL BSA. 5 µL of either extracted DNA from the samples or genomic DNA for the positive control were taken as a template. Thermal cycling conditions were 95 °C for 10 min, followed by 45 cycles of 95 °C for 15 s and 60 °C for 1 min. A positive control of *L. pneumophila* sg 1 DSM 7513 (Leibnitz Institute DSMZ- German Collection of Microorganisms and Cell Cultures, Braunschweig, Germany) genomic DNA diluted to 65.1 pg/µL in the PCR grade water (Promega Corporation, Vienna, Austria) and a non-template control (NTC) PCR grade water (Promega Corporation, Vienna, Austria) were included in all assays. To determine the detection sensitivity of the qPCR, as well as for the generation of standard curves, calibration standards derived from the certified external reference material SRM_LEGDNA_01 ranging from 2.5 × 10^5^ to 2.5 × 10^0^ target gene copies (LEGIONELLES Centres Nationaux de Référence, Lyon, France) were included in every qPCR run. qPCR was performed in a LightCycler 480 II System (Roche Austria GmbH, Vienna, Austria). *Legionella* spp. (*ssrA*) positive samples underwent further testing for *L. pneumophila* (*mip*), and *L. pneumophila* positive samples were further analyzed for *L. pneumophila* sg 1 (*wzm*). Samples were analyzed in duplicates. Samples that tested positive were repeated.

#### 2.4.3. Positive Control

For the positive control, *L. pneumophila* sg 1 DSM 7513 (Leibnitz Institute DSMZ- German Collection of Microorganisms and Cell Cultures, Braunschweig, Germany) genomic DNA was prepared using the DNeasy Blood and Tissue Kit (QIAGEN GmbH, Hilden, Germany), according to the manufacturer’s protocol, with the following modifications: *L. pneumophila* sg 1 DSM 7513 was grown on the BCYE agar plates (VWR International, Vienna, Austria) 36 °C, for seven to 10 days, under CO_2_ pressure (GENbox CO_2_, bioMérieux, Vienna, Austria). Two sterile inoculation loops with 1 µL volume (Greiner Bio-One International GmbH, Kremsmünster, Austria) were mixed into 180 µL buffer ATL, 20 µL proteinase K were added, vortexed, and incubated for 45 min at 56 °C. The quantity and purity of the DNA extracts were measured with a NanoDrop2000 instrument (Thermo Fischer Scientific Inc., Waltham, MA, USA). The extracted genomic DNA was then diluted to 65.1 pg/µL in PCR grade water (Promega Corporation, Vienna, Austria) and included as a positive control in all qPCR assays.

#### 2.4.4. DNA Extraction Control and qPCR Target Specificity Control

In order to test for DNA extraction quality as well as qPCR precision, two one liter samples of sterilized, deionized water were spiked with one LENTICULE DISC each (FEPTU, Public Health England, London, UK). One sample (sample A) contained 1.3 × 10^2^ GU/100 mL *L. bozemanii*, as well as unknown concentrations of *Acinetobacter junii* and *Pseudomonas lundensis*. The other sample (sample B) contained 3.9 × 10^3^ GU/100 mL *L. pneumophila* sg 1, as well as an unknown concentration of *Citrobacter brakii*. Experimental procedure was performed the same as described above for all samples. To further investigate the DNA extraction performance, 100 mL of three separate cooling water samples as well as 100 mL of deionized, sterilized water were each spiked with one LENTICULE DISC containing around 4.12 × 10^4^ CFU *L. pneumophila* NCTC12821 (Culture Collections, Public Health England, London, UK) and the experimental procedure was performed in the same manner as that for other samples. 

#### 2.4.5. Amplification Inhibition Control 

The *egfp* gene was selected as an internal amplification control (IAC), as described by Bliem et al. [14]. A pJET1.2 vector (Thermo Fischer Scientific Inc., Waltham, MA, USA) containing the *egfp* insert was kindly provided by Bliem et al., and was cloned into *Escherichia coli* DH5α competent cells (Thermo Fischer Scientific Inc., Waltham, MA, USA). The plasmid was purified using the QIAprep Spin Miniprep Kit (QIAGEN GmbH, Hilden, Germany) and the quantity and purity of the plasmid was measured using a NanoDrop2000 instrument (Thermo Fischer Scientific Inc., Waltham, MA, USA). The plasmid was diluted to 250 copies/µL in PCR grade water (Promega Corporation, Vienna, Austria) and stored at −20 °C until use. 

#### 2.4.6. Data Analysis

The LightCycler 480 software (Roche Austria GmbH, Vienna, Austria) automatically calculated threshold baselines, slopes, and efficiency, by running the corresponding bacterial gene standard derived from the standard reference material SRM_LEGDNA_01, in a range of 2.5 × 10^5^ to 2.5 × 10^0^ target gene copies. Furthermore, the software automatically calculated mean crossing point (cp) values for replicates, which were used for the final calculations. The cp value of the last detectable standard was set as the limit of detection (LOD) of the qPCR, as the non-template control was not detectable 1 [1].

Statistical analysis was performed using Microsoft Excel and the online program MEDCALC^®^ statistical software [16]. Predictive values were calculated considering the culture method as the reference method for the detection of *Legionella* in environmental water samples. 

## 3. Results

### 3.1. Positive Control and qPCR Precision

For the positive control, *L. pneumophila* sg 1 DSM 7513 genomic DNA with 65.1 pg/µL was used. Cp values for *Legionella* spp. were 29.59 ± 0.21 cycles, for *L. pneumophila*, there were 27.85 ± 0.04 cycles, and for *L. pneumophila* sg there were 1 28.47 ± 0 cycles.

### 3.2. DNA Extraction Control and qPCR Target-Specificity Control

**Figure 1:**
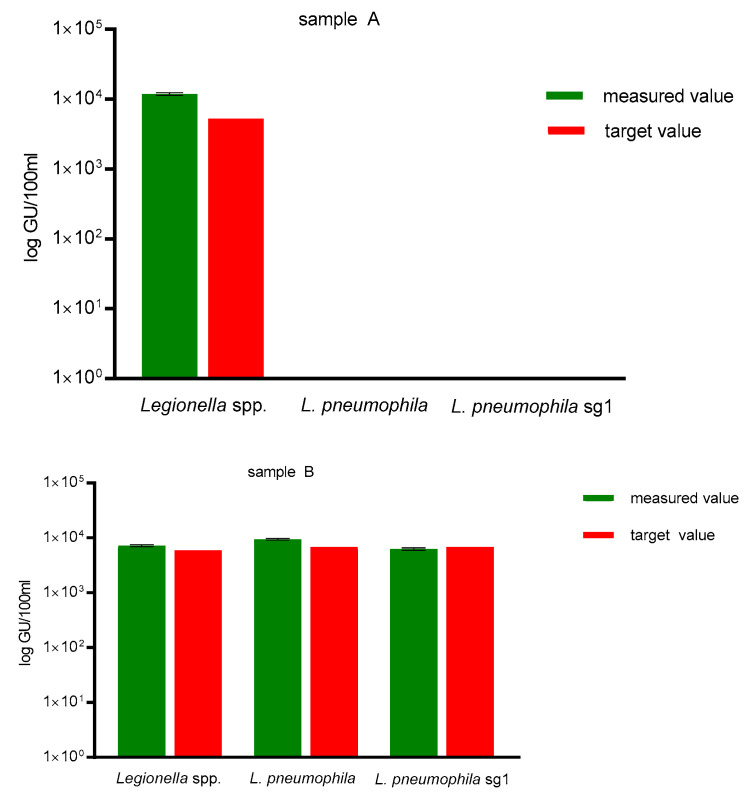
DNA extraction control for all qPCR assays. Measured values obtained for sample A (left graph), containing defined concentrations of 1.3 × 10^2^ GU/100 mL *L. bozemanii* (sample A) showed no amplification for *L. pneumophila* and *L. pneumophila* sg 1, but correct amplification for *Legionella* spp. Measured values for sample B (right graph) showed correct amplification for all three assays, corresponding to the target values of 3.9 × 10^3^ GU/100 mL *L. pneumophila* sg 1 for sample B.

The DNA recovery by the Qiagen PowerWater Kit ranged between 92 to 225% (mean 144%), therefore, exceeding the minimum recovery of 25% recommended in ISO/TS 12869:2012 (see Figure 1). No unspecific amplification for the NTC was observed. 

As a DNA extraction control and qPCR specificity control, sample A contained a defined concentration of 1.3 × 10^2^ GU/100 mL *L. bozemanii* and sample B contained a defined concentration of 3.9 × 10^3^ GU/100 mL *L. pneumophila* sg 1 (samples obtained via FEPTU, Public Health England, United Kingdom). As shown in Figure 1, sample A showed no amplification for *L. pneumophila* and *L. pneumophila* sg 1, but correct amplification for *Legionella* spp. Measured values for sample B showed correct amplification for all three assays, corresponding to the target value of 3.9 × 10^3^ GU/100 mL *L. pneumophila* sg 1 for sample B.

**Figure 2:**
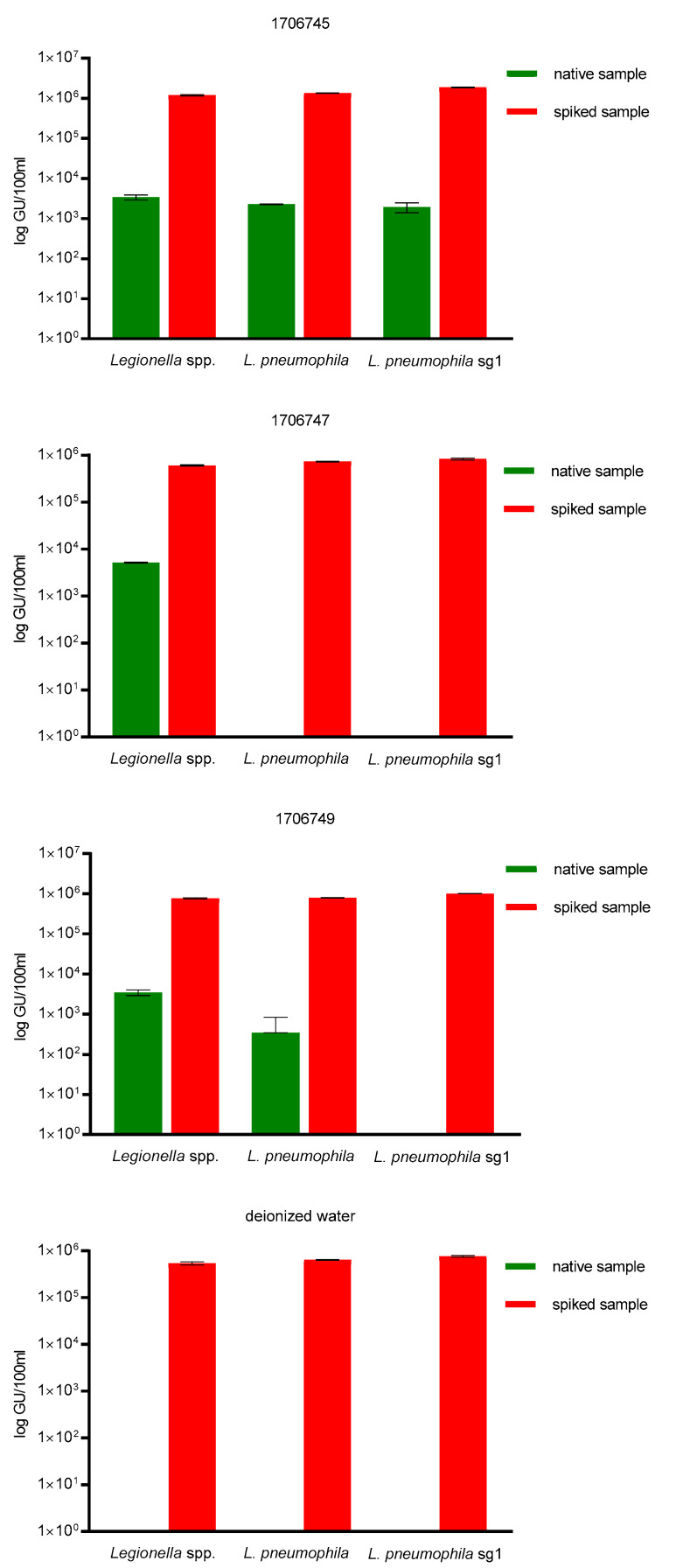
Target specificity control for all three qPCR assays. All three cooling water samples (1706745, 1706747, 1706749) and the sterilized, deionized water had a mean recovery of the ~9.25 × 10^5^ GU/100 mL (±1.45 × 10^5^ GU/100 mL) *L. pneumophila* sg 1 of 100%.

To further determine qPCR target specificity, three cooling water samples as well as sterilized, deionized water were spiked with one LENTICULE DISC containing around ~9.25 × 10^5^ GU/100 mL (±1.45 × 10^5^ GU/100 mL) *L. pneumophila* sg 1. For this purpose, the samples coming from the cooling waters were used, as they represent a difficult sample matrix, due to the potential use of biocides. Water systems that produce aerosols are especially under inspection for Legionella contamination, e.g., cooling towers, hot- and cold-water systems or spa pools, which provide comfortable temperatures for bacterial growth ranging from 20 to 45 °C, due to their heat-exchanging function and thus serve as ‘bacterial amplifiers’, which is why biocides, e.g., bromide and chlorine derivatives or quaternary ammonium compounds might be present in the sample, and could lead to inhibiton of qPCR. No inhibition of qPCR in the spiked samples was observed and the mean recovery of the spiked samples was ~9.25 × 10^5^ GU/100 mL (±1.45 × 10^5^ GU/100 mL) *L. pneumophila* sg 1 for all three qPCR assays was 100% (Figure 2). 

### 3.3. Amplification Inhibition Control

The Qiagen PowerWater Kit allowed for efficient removal of possible inhibitors during DNA extraction, as no inhibition of qPCR in the samples was observed and the samples were therefore used undiluted in the qPCR assays. Mean cp values for the IAC were 30.63 ± 0.21 cycles. Samples were considered to be inhibited if the ct values shifted higher than 2 cycles, as compared to the IAC in the NTC, in this case, the samples were repeated diluted.

### 3.4. Linearity and Limits of Detection of qPCR

Quantification of *Legionella* spp. as well as *L. pneumophila* and *L. pneumophila* sg 1 was linear between the 2.5 × 10^0^ and 2.5 × 10^5^ GU/reaction. The LightCycler 480 software (Roche Austria GmbH, Vienna, Austria) automatically calculated reaction efficiencies (E = 10^−1/slope^), which ranged from 1.954 to 2.11 for all assays, with the expected systematical error (2^n^/E^n^ − 1) × 100 staying below 0.0345 for all three qPCR assays. LOD was 2.5 × 10^0^ GU/reaction, corresponding to an LOD of 5 × 10^1^ GU/100 mL for all three assays used. No amplification of the NTC was observed. 

### 3.5. Limits of Detection for Culture

For culture performed according to ISO 11731:2017, the LOD was 1 CFU/100 mL for the culture Matrix A, whereas for Matrix B, the LOD was 20 CFU/100 mL. The LOD of the culture methods was dependent on the volume filtrated for either of the Matrix procedures, which was 1 as well as 100 mL for Matrix A; or for Matrix B, from a filtration volume of 100 mL that was recovered in 5 mL, 0.25 mL were plated.

### 3.6. Comparison of Culture Matrix A and B 

Thirteen water supply samples (20.31%) tested positive for *L. pneumophila* contamination, with one of those samples (7.69%) testing positive via Matrix B and nine samples (69.23%) testing positive via Matrix A. In combination, two samples (15.83%) tested positive for *L. pneumophila* using Matrix A as well as Matrix B. 

### 3.7. Culture (ISO 11731:2017) and qPCR (ISO/TS 1286:2012) in Comparison for Environmental Water Samples

Culture according to ISO 11731:2017 and qPCR based on ISO/TS 1286:2012 were carried out for environmental water samples (*n* = 64) analyzed routinely for *Legionella* contamination. Of the analyzed samples in this study, 31 samples (48.44%) were negative for *Legionella* spp. using both culture and qPCR. qPCR detected *Legionella* spp. via the *ssrA* gene in 29 out of 64 samples (45.31%). 

For samples with a presumed low burden of the accompanying microbial flora (*n* = 46), *L. pneumophila* sg 2–15 was detected using culture Matrix A in nine samples (19.57%), whereas none were positive using culture Matrix B. qPCR found *L. pneumophila* DNA contamination in four samples (8.70%). There were eight culture positive-qPCR negative samples (17.39%) and three qPCR positive-culture negative samples (6.52%) (see Table 3). This results for samples with an expected low burden of accompanying microbial flora in a PPV of 75.00%, and an NPV of 82.22% for the *mip*-based qPCR (see Table 4).

For samples with a presumed high burden of accompanying microbial flora (*n* = 18), *L. pneumophila* sg 2–15 was detected using culture Matrix A in two (11.11%) samples, whereas only one was positive only by culture Matrix B (5.56%). qPCR found *L. pneumophila* DNA contamination in five samples (27.78%) samples. There were no culture positive-qPCR negative samples and three qPCR positive-culture negative samples (16.67%) (see Table 3). This results for samples with an expected high burden of accompanying microbial flora in a PPV of 40.00%, and an NPV was 100.00% for the *mip*-based qPCR (see Table 4). 

For samples with a presumed low burden of accompanying microbial flora (*n* = 46), culture did not detect *L. pneumophila* sg 1 in any sample, which corresponded with qPCR results and did not find *L. pneumophila* sg 1 DNA contamination, and therefore no PPV could be predicted. 

For samples with a presumed high burden of accompanying microbial flora (*n* = 18), culture detected *L. pneumophila* sg 1 in one sample (5.56%), and qPCR found *L. pneumophila* sg 1 DNA in five samples (27.78%). There were no culture positive-qPCR negative samples and four qPCR positive-culture negative samples (22.23%). This resulted in a PPV of 17.00% and a NPV of 100.00% for the *wzm*-based qPCR. 

qPCR detected *Legionella* spp. via the *ssrA* gene in concentrations ranging 4.41 × 10^2^ GU/100 mL to 1.15 × 10^6^ GU/100 mL in 29 samples, *L. pneumophila* via the *mip* gene in nine samples, in concentrations ranging from 6.56 × 10^2^ GU/100 mL to 3.88 × 10^5^ GU/100 mL and in *L. pneumophila* sg 1 via the *wzm* gene, in concentrations ranging from 1.32 × 10^2^ GU/100 mL to 1.47 × 10^5^ GU/100 mL in five samples. 

On the other end, the culture identified *L. pneumophila* in concentrations ranging from 2 CFU/100 mL to 1 × 10^2^ CFU/100 mL for Matrix A in eleven samples. For *L. pneumophila* sg 1, the culture detected one sample using only Matrix B at and detected concentrations of 7.2 × 10^2^ CFU/100 mL.

## 4. Discussion

qPCR is considered to be a fast and convenient method for rapid *Legionella* detection from environmental water samples, providing a high specificity for the amplified target. However, qPCR comes with its own set of limitations, one being the possible presence of inhibiting substances in the samples of interest (such as humic acids or ferric ions), and the other being the detection of not only viable, but also dead bacteria and bacterial cells being in the viable-but-non-culturable (VBNC) state [13]. As of now, it is difficult to compare qPCR results with those obtained by culture, as the qPCR results are expressed in genomic units (GU) and the culture results are in colony forming units (CFU), which is the format given in most guideline documents. There are some calculations available, such as Lee et al. (2011), who reported that qPCR results are four- to five-fold higher than culture results. Yaradou et al. (2007) also described five-fold higher qPCR results than the culture and Ditommaso et al. (2015) proposed a conversion factor of 28-fold from qPCR to culture [10,12,17]. However, no conversion factor between GU and CFU is yet implemented into the guideline documents and the standard methods [15]. Authors such as Hamilton et al. (2019) suggest the necessity of additional datasets for this conversion factor for statistical models before implementation of such a conversion factor [18].

As previously reported, DNA extraction efficiencies and PCR inhibitor removal are dependent on the extraction and purification method used [14]. In this study, we used a commercially available kit for DNA extraction for all different water samples, which provided good DNA recovery as well as inhibitor removal and allowed the undiluted use of DNA extracts during qPCR (see Figure 1).

Our study confirmed numerous previous investigations [2,4,9,12,19,20,21,22] that showed more qPCR positive than culture positive results, which could be explained by the difficulty of *Legionella* cultivation, the existence of cells being in the VBNC state, dead bacteria, and a higher sensitivity of qPCR. This, in turn, leads to a conceivably low PPV of qPCR for culture results [4]. As alternative solutions, recent studies propose the use of propidium monoazide or ethidium monoazide nucleic acid dyes, prior to DNA extraction for qPCR, to inhibit DNA amplification from dead bacterial cells to further improve qPCR comparability with culture methods [10]. Other methods to detect viable and virulent *Legionella* at low concentrations might be amoebic co-culture prior to qPCR, as their ability to invade and multiply within *Acanthamoeba* might indicate their pathogenicity [23], or immunomagnetic separation, which is a method not affected by competing microbial flora or inhibitors present in the sample, and can show success of biocide treatment, as it detects only viable *Legionella* and would, therefore, be very useful for risk assessment and water safety plans [24].

In our study, culture-negative–qPCR-positive occurred with a rate of 17.39% in samples with an expected low accompanying microbial flora, with all samples giving results of 2 CFU/100 mL in the culture. Other studies also observed the phenomenon that samples with a low CFU/100 mL count might give qPCR-negative results, and these authors imply that samples with a low concentration have little chance of causing disease [25]. From a public health view, the Austrian standard ÖNORM B 5019:2020-3 *Hygienerelevante Planung, Ausführung, Betrieb, Überwachung und Sanierung von zentralen Trinkwasser-Erwärmungsanlagen* states an alert level of 10^4^ CFU/L, which requires immediate sanitation of the site, and this concentration can be reliably detected in our study, which goes in line with the guidelines in other countries such as Spain, Denmark, or Italy, which also apply an alert level of 10^4^ CFU/L [26]. However, so far, no direct relationship was established between *Legionella* load and disease, but a quantitative microbial risk assessment calculated by Hamilton et al. (2019), estimated a critical concentration of 10^3^ CFU/L [18]. Disease and finding *L. pneumophila* sg 1, no matter if via qPCR or culture, is an indicator that further investigation and preventive measures for rehabilitation of the affected source might be advisable [4,27].

As previously recognized [5,8], the serotyping of *L. pneumophila* isolates as well as the culture methods in ISO 11731:2017 introduce a bias towards isolation of *L. pneumophila* and possibly neglects other *Legionella* spp. present in the sample, which was also shown to be especially prevalent in water samples taken from sources with temperatures below 37 °C [4]. Similar to other studies, all isolated strains in this study were *L. pneumophila*, and 92.31% of the *L. pneumophila* isolates belonged to serogroups 2–14, which accounted for about 15–20% of the community-acquired Legionellosis cases [28]. However, *L. pneumophila* is thought to be the causative agent in 95% of all cases of Legionnaires disease worldwide and *L. pneumophila* sg 1 for about 70% of cases in Europe [2,4]. The qPCR assay for the detection of *L. pneumophila* sg 1 proved to be reliable and specific in detection for samples with an expected high burden of accompanying microbial flora, such as cooling towers, etc.

As repeatedly stated in previous studies, one of the pitfalls of culture-based *Legionella* detection is the long turnover time for the generation of results (up to ten days) [2,4,7,9]. In our study, qPCR shows a high NPV for *L. pneumophila* sg 1 for samples, independent of their presumed accompanying microbial flora, which indicates a high reliability of qPCR for a possible combined use of qPCR and culture. In an outbreak setting, where detection of *L. pneumophila* sg 1 is time-sensitive, qPCR could be done in as little as one day, which in turn could lead to faster public alert and preventive measures could be taken quickly, and the culture could still be performed on positive samples for confirmation and strain isolation, as also suggested in the literature [2,4,9].

With regards to the updated ISO 11731:2017, internal validation of the method in our laboratory showed that Matrix B could lead to significantly lower results in comparison with Matrix A, as the harsh treatments used in Matrix B not only eliminate the accompanying microbial flora, but also low numbers of *Legionellae*. In addition to the filtered volume of 100 mL required in in most guidelines, we further recommend the additional filtration of 1 mL for Matrix A to assess the competing microbial flora and prevent plate overgrowth, as often the filter of the 100 mL filtration sample could be overgrown. It might also be useful to inspect the plates earlier than the ten days incubation for filter overgrowth prevention, as ISO 8199 *Water quality—General requirements and guidance for microbiological examinations by culture* defines the upper LOD as 80 CFU per filter, which could be exceeded if there is a high microbial flora present in the sample, and the filter is directly placed onto the GVPC agar after filtration. According to this, inspecting the agar plates earlier on day 2, 5, 7, and 10, could help detect *Legionella* before the plates are overgrown. 

The limitations of our study lie in the low numbers of samples included in the study as well as the low volume filtrated for qPCR, which could have led to more qPCR-negative results. However, this limitation could be easily overcome by implementing the collection of larger (or multiple smaller flasks) volumes into the standard protocols. The strength of the study lies in the use of the different matrices described in the ISO standard to routine water samples and the additional evaluation by qPCR.

## 5. Conclusions

In conclusion, because of the excellent NPV of all qPCR assays used, especially for samples with a presumed high microbial burden, we strongly suggest implementation of qPCR as a method for screening out *Legionella*-negative samples, prior to starting labor-intensive culture methods. 

## Figures and Tables

**Table 1:** Sample handling for *Legionella* detection by culture, based on ISO 11731:2017.

Decision Matrix for Sample Handling				
	Matrix A		Matrix B	
intended purpose	low concentration of accompanying microbial flora		high concentration of accompanying microbial flora	
Method	Method A	Method B	Method B	Method C dilution 1:10
sample handling	no treatment	no treatment	no treatment	no treatment
		acid treatment	acid treatment	acid treatment
		heat treatment	heat treatment	heat treatment
culture medium	BCYE ^1^	BCYE ^1^, GVPC ^2^	GVPC ^2^	GVPC ^2^

^1^ BCYE agar plates, ^2^ GVPC agar plates.

**Table 2:** qPCR conditions.

qPCR		Oligonucleotide Sequence (5′-3′)		Reference
***Legionella* spp. qPCR**				
**primer forward:**		GGCGACCTGGCTTC		[2]
**primer reverse:**		GGTCATCGTTTGCATTTATATTTA		
**probe:**		FAM-ACGTGGGTTGCAA-MGBNFQ ^1^		
**product size:**	101 bp			
***Legionella pneumophila* qPCR**				
**primer forward:**		TTGTCTTATAGCATTGGTGCCG		[2]
**primer reverse:**		CCAATTGAGCGCCACTCATAG		
**probe:**		CY5-CGGAAGCAATGGCTAAAGGCATGCA-BHQ1 ^2^		
**product size:**	115 bp			
***Legionella pneumophila* serogroup 1 qPCR**				
**primer forward:**		TGCCTCTGGCTTTGCAGTTA		[2]
**primer reverse:**		CACACAGGCACAGCAGAAACA		
**probe:**		HEX-TTTATTACTCCACTCCAGGCGAT-BHQ1 ^2^		
**product size:**	70 bp			
***Internal Amplification Control***				
**IAC primer forward:**		GACCACTACCAGCAGAACAC		[4]
**IAC primer reverse:**		GAACTCCAGCGGACCATG		
**probe:**		HEX/CY5-ACGTGGGTTGCAA-BHQ1 ^2^		
**product size:**	132 bp			

^1^ Minor groove bender non-fluorescent quencher and ^2^ Black Hole Quencher 1^®^ (Eurofins Genomics, Ebersberg, Germany).

**Table 3:** Positive samples with low and high burden of accompanying microbial flora from qPCR and culture for *L. pneumophila* and *L. pneumophila* sg 1, established from the *L. pneumophila* qPCR for the *mip* gene and the *L. pneumophila* sg 1 qPCR for the *wzm* gene.

	Comparison of qPCR and Culture	
	low burden of accompanying microbial flora (*n* = 46)	
No. of positive samples	culture	qPCR
*Legionella pneumophila* (*mip*)	9	4
	(8 culture positive–qPCR negative)	(3 qPCR positive–culture negative)
*Legionella pneumophila* sg1 (*wzm*)	0	0
	**high burden of accompanying microbial flora (*n* = 18)**	
No. of positive samples	culture	qPCR
*Legionella pneumophila* (*mip*)	2	5
		(3 qPCR positive–culture negative)
*Legionella pneumophila* sg1 (*wzm*)	1	5
		(4 qPCR positive–culture negative)

**Table 4:** Predictive values of qPCR for *L. pneumophila* and *L. pneumophila* sg 1 culture results, established from the *L. pneumophila* qPCR for the *mip* gene and the *L. pneumophila* sg 1 qPCR for the *wzm* gene, in comparison with the culture for samples with a low and high burden of the accompanying microbial flora.

Predictive Value in %				
qPCR	Culture			
	Low Burden of Accompanying Microbial Flora (*n* = 46)		High Burden of Accompanying Microbial Flora (*n* = 18)	
	**PPV ^1^**	**NPV ^2^**	**PPV ^1^**	**NPV ^2^**
***Legionella pneumophila* (*mip*)**	75.00	82.22	40.00	100.00
***Legionella pneumophila* sg1 (*wzm*)**	not predictable	100.00	17.00	100.00

^1^ Positive Predictive Value; ^2^ Negative Predictive Value.

## Data Availability

Data sharing not applicable.

## References

[1] Berjeaud J.M., Chevalier S., Schlusselhuber M., Portier E., Loiseau C., Aucher W., Lesouhaitier O., Verdon J. (2016). Legionella pneumophila: The paradox of a highly sensitive opportunistic waterborne pathogen able to persist in the environment. Front. Microbiol..

[2] Collins S., Jorgensen F., Willis C., Walker J. (2015). Real-time PCR to supplement gold-standard culture-based detection of legionella in environmental samples. J. Appl. Microbiol..

[3] Lucas C.E., Taylor T.H., Fields B.S. (2011). Accuracy and precision of legionella isolation by US laboratories in the ELITE program pilot study. Water Res..

[4] Fisher K.E., Wickenberg L.P., Leonidas L.F., Ranz A.A., Habib M.A., Buford R.M., McCoy W.F. (2020). Next day legionella PCR: A highly reliable negative screen for legionella in the built environment. J. Water Health..

[5] World Health Organization (2007). Legionella and the Prevention of Legionellosis.

[6] Ditommaso S., Giacomuzzi M., Ricciardi E., Zotti C.M. (2015). Viability-qPCR for detecting legionella: Comparison of two assays based on different amplicon lengths. Mol. Cell Probes..

[7] Whiley H., Taylor M. (2016). Legionella detection by culture and qPCR: Comparing apples and oranges. Crit. Rev. Microbiol..

[8] Boulanger C.A., Edelstein P.H. (1995). Precision and accuracy of recovery of legionella pneumophila from seeded tap water by filtration and centrifugation. Appl. Environ. Microbiol..

[9] Collins S., Stevenson D., Walker J., Bennett A. (2017). Evaluation of legionella real-time PCR against traditional culture for routine and public health testing of water samples. J. Appl. Microbiol..

[10] Lee J.V., Lai S., Exner M., Lenz J., Gaia V., Casati S., Hartemann P., Lück C., Pangon B., Ricci M.L. (2011). An international trial of quantitative PCR for monitoring legionella in artificial water systems. J. Appl. Microbiol..

[11] Gensberger E.T., Polt M., Konrad-Koszler M., Kinner P., Sessitsch A., Kostic T. (2014). Evaluation of quantitative PCR combined with PMA treatment for molecular assessment of microbial water quality. Water Res..

[12] Yaradou D.F., Hallier-Soulier S., Moreau S., Poty F., Hillion Y., Reyrolle M., André J., Festoc G., Delabre K., Vandenesch F. (2007). Integrated real-time PCR for detection and monitoring of legionella pneumophila in water systems. Appl. Environ. Microbiol..

[13] Smith C.J., Osborn A.M. (2009). Advantages and limitations of quantitative PCR (Q-PCR)-based approaches in microbial ecology. FEMS Microbiol. Ecol..

[14] Bliem R., Schauer S., Plicka H., Obwaller A., Sommer R., Steinrigl A., Alam M., Reischer G.H., Farnleitner A.H., Kirschner A. (2015). A novel triplex quantitative PCR strategy for quantification of toxigenic and nontoxigenic vibrio cholerae in aquatic environments. Appl. Environ. Microbiol..

[15] Toplitsch D., Platzer S., Pfeifer B., Hautz J., Mascher F., Kittinger C. (2018). *Legionella* detection in environmental samples as an example for successful implementation of qPCR. Water.

[16] MedCalc Software bvba MedCalc^®^ Easy to Use Statistical Software. Diagnostic Test Evaluation Calculator. https://www.medcalc.org/calc/diagnostic_test.php.

[17] Ditommaso S., Ricciardi E., Giacomuzzi M., Arauco Rivera S.R., Zotti C.M. (2015). Legionella in water samples: How can you interpret the results obtained by quantitative PCR?. Mol. Cell Probes..

[18] Hamilton K.A., Hamilton M.T., Johnson W., Jjemba P., Bukhari Z., LeChevallier M., Haas C.N., Gurian P.L. (2019). Risk-based critical concentrations of legionella pneumophila for indoor residential water uses. Environ. Sci. Technol..

[19] Caicedo C., Rosenwinkel K.H., Exner M., Verstraete W., Suchenwirth R., Hartemann P., Nogueira R. (2019). Legionella occurrence in municipal and industrial wastewater treatment plants and risks of reclaimed wastewater reuse: Review. Water Res..

[20] Kirschner A.K.T. (2016). Determination of viable legionellae in engineered water systems: Do we find what we are looking for?. Water Res..

[21] Bonetta S., Bonetta S., Ferretti E., Balocco F., Carraro E. (2010). Evaluation of legionella pneumophila contamination in italian hotel water systems by quantitative real-time PCR and culture methods. J. Appl. Microbiol..

[22] Guillemet T.A., Levesque B., Gauvin D., Brousseau N., Giroux J.P., Cantin P. (2010). Assessment of real-time PCR for quantification of legionella spp. in spa water. Lett. Appl. Microbiol..

[23] Edagawa A., Kimura A., Miyamoto H. (2019). Investigations on contamination of environmental water samples by legionella using real-time quantitative PCR combined with amoebic co-culturing. Biocontrol. Sci..

[24] Diaz-Flores A., Montero J.C., Castro F.J., Alejandres E.M., Bayón C., Solís I., Fernández-Lafuente R., Rodríguez G. (2015). Comparing methods of determining legionella spp. in complex water matrices. BMC Microbiol..

[25] Jinadatha C., Stock E.M., Miller S.E., McCoy W.F. (2018). Environmental validation of legionella control in a VHA facility water system. Infect. Control. Hosp. Epidemiol..

[26] Krojgaard L.H., Krogfelt K.A., Albrechtsen H.J., Uldum S.A. (2011). Detection of legionella by quantitative-polymerase chain reaction (qPCR) for monitoring and risk assessment. BMC Microbiol..

[27] National Academies of Sciences, Engineering, and Medicine, Health and Medicine Division, Division on Earth and Life Studies, Board on Population Health and Public Health Practice, Board on Life Sciences, Water Science and Technology Board, Committee on Management of Legionella in Water Systems, National Academies Press (US) (2019). Quantification of legionnaires’ disease and *legionella*. Management of Legionella in Water Systems.

[28] Borella P., Montagna M.T., Stampi S., Stancanelli G., Romano-Spica V., Triassi M., Marchesi I., Bargellini A., Tato D., Napoli C. (2005). Legionella contamination in hot water of italian hotels. Appl. Environ. Microbiol..

